# Treatment of Mesenteric Glandular Affections

**Published:** 1842-06

**Authors:** Charles Clay

**Affiliations:** Manchester


*Treatment of Mesenteric Glandular Affections.* By Dr. Charles Clay, Manchester.—Under the name of marasmus, and as an affection of childhood, diseased mesenteric glands are very common, almost daily coming under the notice of medical practitioners, *particularly in manufacturing districts*, where laxity of fibre and much constitutional debility prevail, and where the habit, diet, &c., contribute considerably to its encouragement. But as a disease of puberty or adult growth, comparatively little is known; yet it is certain that a disease strictly analogous to the marasmus of childhood is very prevalent, much more so, indeed, than is generally admitted, and annually carries off many whose deaths are often attributed to very different causes, even in other than manufacturing districts. Three-fourths of the common cases of atrophy are attributable to glandular obstruction only. From the many cases that I have observed during twenty years practice, and in the treatment of which I have been engaged, a few practical observations with respect to it may not be unacceptable, particularly as there are few cases that call forth more patience from the medical attendant, and certainly none that are productive of greater disappointment, rendering it in the highest degree necessary to make a correct diagnosis, which having been made, a long and steady perseverance in remedial measures is to be pursued. I say a long and steady perseverance, because the nature of the case particularly requires it, not only on the part of the medical attendant, but also of the patient. In all cases of long-continued disease there is an aptness to run from one practitioner to another, vainly seeking that immediate relief which cannot possibly be given. If a correct diagnosis be once formed, and the slightest benefit obtained, every confidence should be placed in the party, and perseverance should be the motto of the invalid.

The treatment of mesenteric affections is so often confounded with that of atrophy from other causes, that great errors have arisen, which it is the more necessary to point out. No gland can be enlarged or indurated by disease without obstructing the operations of nature, for which those glands were especially formed; it is evident, therefore, when such indurations are ascertained to have taken place, our attempts at relief should be directed to the chief cause of mischief. Where glandular obstructions exist, it is evident that the quantity of chyle must be limited in proportion to the extent of diseased structure, and in the same proportion the system suffers from emaciation, not receiving the supply of nutriment sufficient to maintain the system in its former healthy state; consequently, this disease is found in every stage, from the slightest obstruction to the entire obliteration of the glandular action; no chyle poured into the blood, the system is emaciated in the extreme; the limbs or parts most distant from the centre of circulation become œdematous, and death immediately follows.

If, then, glandular obstruction be the diagnosis, what is to be done? This leads me to make a remark or two on some of the general axioms of treatment pursued in such cases, with a view to point out their errors, and substitute a plan that the writer has often pursued with considerable advantage.

Dr. Thomas observes, "That in all cases of atrophy the patient should make use of food that is nutritive and easy of digestion, and it should be taken frequently, but in small quantities at a time." I fully agree with him as to the kind of food, but maintain that to feed an atrophic patient *frequently* is a very mischievous doctrine, calculated to increase rather than lessen the evil: that the whole of the alimentary canal is much deranged in atrophic cases is certain, and the greatest caution is required in the selection of such food as will not require too great an effort for the digestive function; but for that organ to be continually stimulated by constantly taking food is decidedly injurious, the stomach requiring rest from its usual operations as much as any other organ in the body. The repose of the digestive function is necessary to the well-being of an invalid, and in none more so than in atrophic cases, when it is evident, however much chyme may be formed, no more chyle can pass into the system (in consequence of the indurated glands), if the patient be eating every hour in the day, or if only allowed to indulge three times in twenty-four hours; there can be then no advantage in debilitating digestion by frequent meals, whilst the disadvantages must be apparent to every one on the slightest reflection, viz., it increases general debility, and prevents the due operation of medicine calculated to resolve the existing indurations and consequent obstruction in the mesenteric glands. Impaired digestive function is the consequence, *and not the cause,* of the prevailing disease in this case. The real disease should be primarily attacked with the necessary caution of not impairing the powers of digestion further; it will then be evident that all those symptoms necessarily arising from such causes must give way as the cause which provoked them vanishes. The too frequent exhibition of, and too much dependence on, powerful tonics, appears to me contrary to the diagnosis of mesenteric glandular affections, for it must be useless to waste time in the endeavor to improve the function of the stomach by tonic medicines, which, though considerably impaired, *is capable of digesting more food* than can be converted into chyle. The symptoms of indigestion we so often find as attendants of glandular obstruction, are often increased to a very considerable degree by the ill-advised and constant taking-food system.

Very many cases of this description are treated in respect to the miseries of indigestion, whilst the glandular affection is either totally lost sight of, or treated merely as a secondary matter of minor importance, whereas the very reverse ought to be pursued. The constant stimulus kept up by tonics, in an emaciated system (extremely sensitive and easily excited), counteracts every effort at reducing glandular induration; and this is rendered still more injurious by wines and other stimulating drinks which have been recommended. Such a mode of treatment might be adopted in the common forms of scrofula with less objection than in mesenteric affection, but I must confess *even in those* I never perceived stimulants attended with any good effect. In atrophic cases, however, they are highly pernicious, although they are esteemed by some as scrofulous affections. When œdema takes place, diuretics have been advised; but as this feature is one pointing out the inevitably fatal termination of the case, such means can only hasten the event, being only another drain on the already debilitated system, without adding anything to the supply.—Should any means adopted prove successful in restoring the glands to a healthy action by resolving the existing induration and restoring them to their original size, then there can be no mistake in the exhibition of tonics, freely adopting every means for restoring the physical powers of the system.

The treatment which I have found most effectual, and which I do not advance on mere theory, but from twenty year's close observation, is the use of a medicine that is generally allowed to be almost a specific in diseases of the glandular system, and that in doses so small as not to excite the disturbance of the digestive organs, combining such means with milder tonics just sufficient to keep the system from sinking any lower, without any anxiety for increasing the physical powers, until the indurated glands may have been restored; under such circumstances I commence by giving the following:—R. Tincture of iodine, gtts. xxx.; Fowler's solution of arsenic, gtts. xxv.; infusion of colombo or gentian, ℥ vi. M. Let one-sixth be taken three times a day.

As it sometimes happens that the solution of arsenic produces pains in the head, I occasionally omit it in the mixture for the space of two or three days, after which it is resumed. By persevering some time steadily with this mixture, I have found the worst cases much ameliorated, and life considerably lengthened, whilst many have been entirely restored to health; but as glandular resolution is of itself an extremely slow process, so it requires both perseverance and confidence on the part of the invalid, and great patience from the medical attendant. It is also necessary in the progress of cure to affect the system very slightly with mercury once or twice, and in some cases of extensive disease of long standing even three times with great advantage, by which means the absorbent vessels are stimulated into freer action, and the effects of the iodine seem to be improved by it. When it is requisite to give mercury, I prefer affecting the system as rapidly as possible by very small doses of calomel very often repeated, as—R. Calomel, grs. ij.; crumb of bread, enough to make twenty-four pills. Take one every hour until the mouth is affected.

The advantage in this is, that the desired effect is frequently produced within twenty-four hours, when the iodine mixture can be resumed (which it is necessary to omit while the effect is being produced). This plan, if strictly attended to, is one that I can recommend with confidence as a safe and effectual one, applicable to every case of glandular induration, and unsuccessful only in cases too long neglected, where the action of the glands is almost entirely obliterated. The diet should be strictly such as to afford the greatest quantum of nourishment with the least possible exertion of the stomach, to be well masticated, mixed with as little fluid as possible (with the exception of milk), and particularly to avoid those of a highly stimulating character, such as wines, spirits, and fermented liquors: to let a space of at least six hours elapse between taking food, and even then the stomach should not be overloaded. These rules are imperative to the well-being of the patient. Exercise should be of the gentlest description; why horse exercise should be so highly spoken of by many, I cannot conceive; in may instances I have seen it the very reverse of gentle; only fancy a weak, emaciated female tugging at the reins, and urging forward a stupid, rough-paced animal with an exertion highly injurious; unless the adviser would go farther and say the kind of horse he recommends, he might as well send his patient to the treadmill: unless, then, the horse is a very suitable one, I am convinced the patient would progress better without such exercise. Where it can be procured, and weather permitting, an airing in an open carriage, or a gentle walk, is to be preferred; if, on the contrary, the weather is unfit, a swing rocking horse, or exercising chair, are very good substitutes; the mind to be kept cheerful, free from extraordinary excitements, occupied rather on pleasant trifles than on subjects requiring reflection. The atrophic cases of manufacturing districts, however, have but little comfort at command; still I have seen many restored under almost every disadvantage, and am anxious the plan should have a more general application, that its merit may be fully and fairly tested.—*London Lancet.*

